# Supplementary material for: Nuclear envelope deformation controls cell cycle progression in response to mechanical force
Source: EMBO Rep. 2019 Aug 1;20(9):e48084. doi: 10.15252/embr.201948084 (PMC6726894; doi:10.15252/embr.201948084)
Supplement: Supplementary file 1 — Expanded View Figures PDF [file EMBR-20-e48084-s001.pdf]

Expanded View Figures

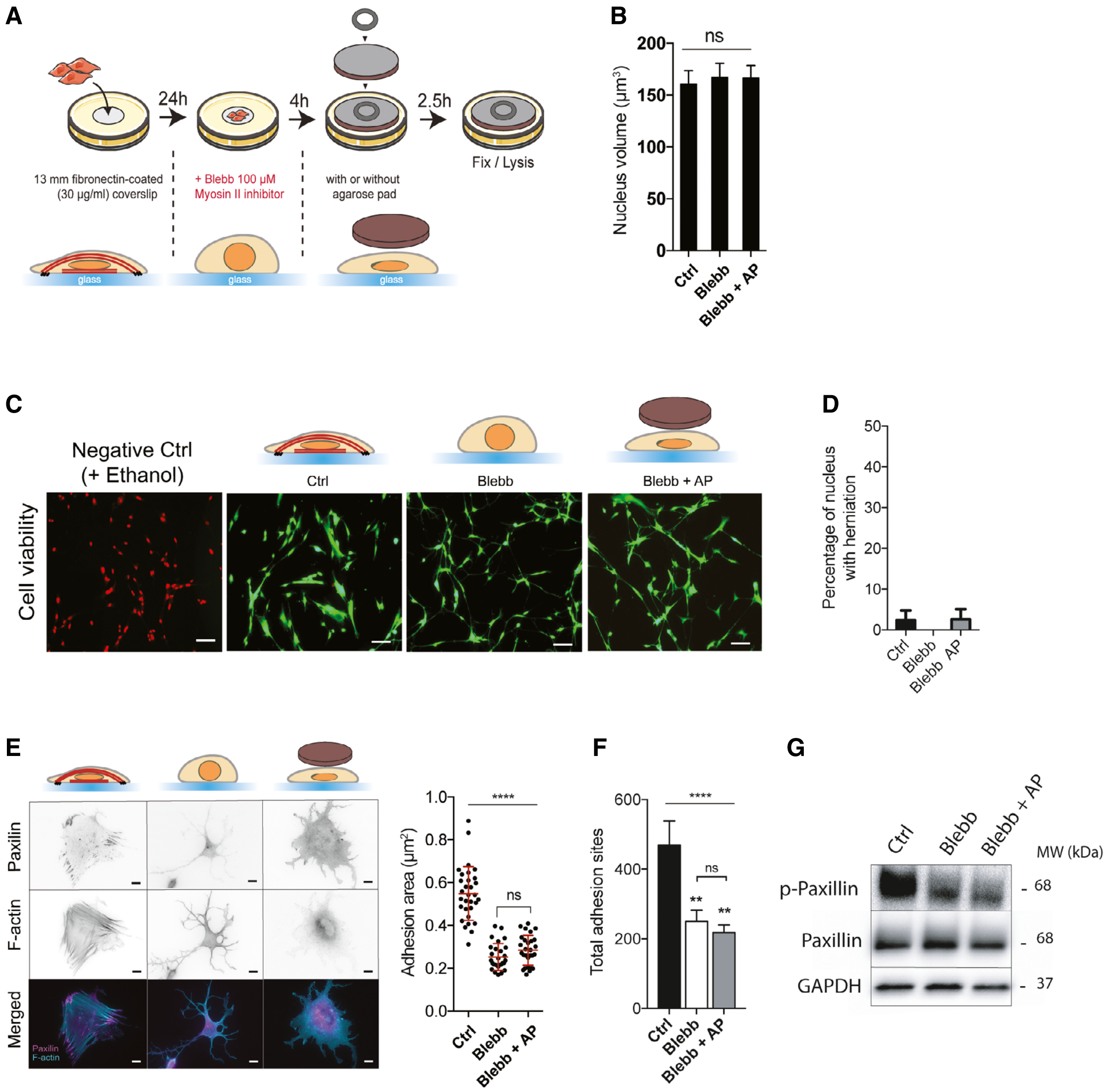

Figure EV1. Agarose pad-mediated nuclear flattening.

A MRC5 cells were cultured on a 13-mm fibronectin-coated glass coverslip (for IF) or a 35-mm fibronectin- coated dish (for biochemistry) during 24 h. The medium is replaced with a fresh medium containing blebbistatin (100 μM) for 4 h. Then, an agarose pad (AP) is gently applied on cells. A 32-mm coverslip with a 425 mg washer is used to maintain the agarose pad. 2.5 h later, cells are fixed using 3.7% PFA (IF) or directly lysed with a Laemmli lysis buffer.

B DNA staining was used to quantify nuclear volume in the indicated conditions. Data are presented as mean ± s.e.m. (n = 37 minimum from two independent experiments, ns: not significant, one-way ANOVA).

C Representative images of MRC5 cells assessed for cell viability using calcein/AM (green) and ethidium homodimer (Red) in Ctrl, Blebb, and Blebb + AP conditions. Ethanol was used to induce cell apoptosis (negative Ctrl).

D Percentage of cells with nuclear herniation. Data are presented as mean ± s.e.m. (n = 40 minimum from two independent experiments).

E, F Representative cells stained for actin-F (cyan) and focal adhesions (paxillin—magenta) in Ctrl, Blebb, and Blebb + AP conditions. Scale bar = 10 μm. Quantifications of (e) adhesion areas using paxillin staining (n = 28 from three independent experiments, ****$P < 0.001$ one-way ANOVA—Tukey's multiple comparisons post-test) and (f) total adhesion sites using p-Tyr antibody (n = 10, **$P < 0.01$, ****$P < 0.001$ one-way ANOVA—Tukey's multiple comparisons post-test). Data are presented as mean ± s.e.m.

G Immunoblots of p-paxillin (Y118), paxillin, and GAPDH for Ctrl, Blebb, and Blebb + AP conditions.

© 2019 The Authors

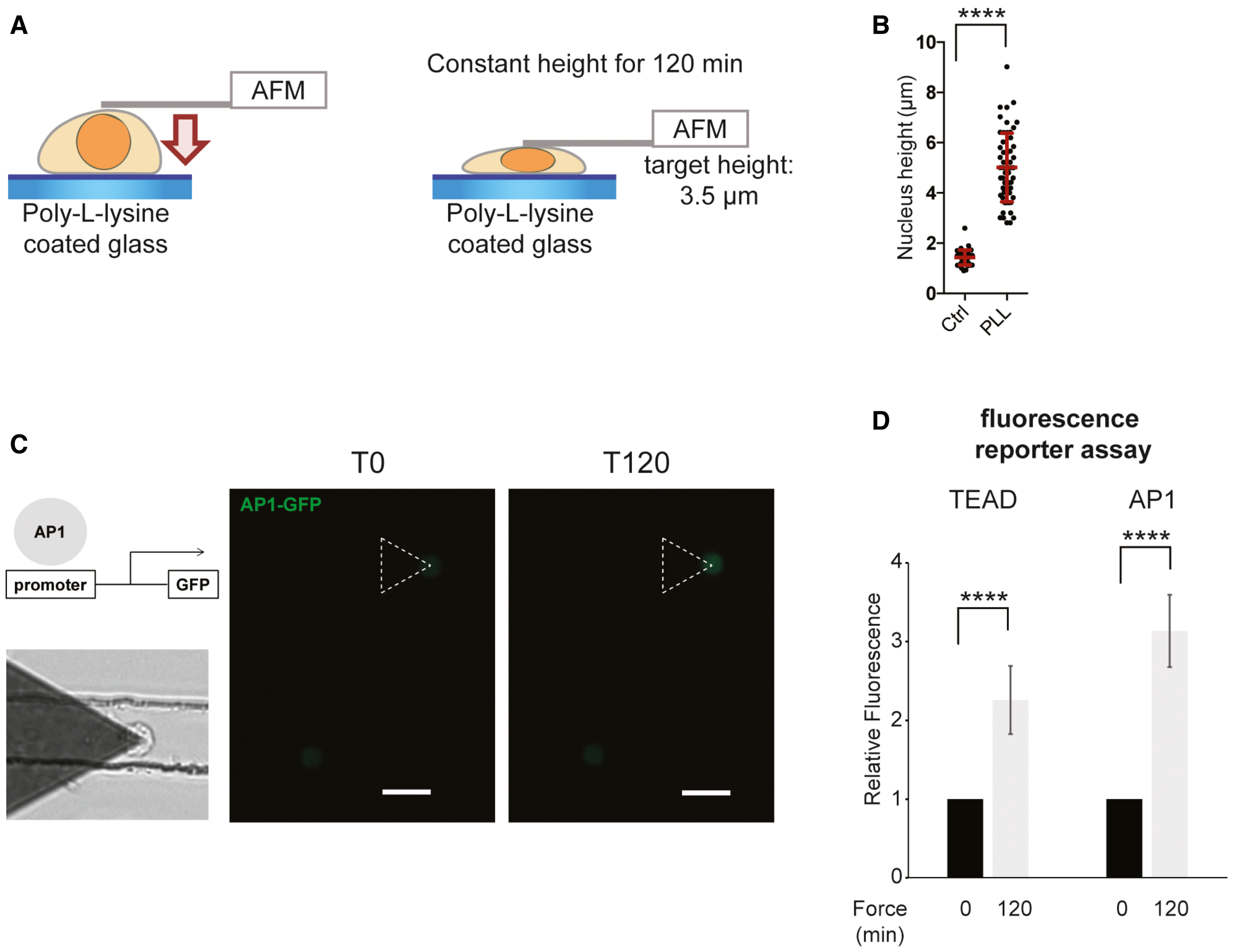

Figure EV2. TEAD and AP1 activation in response to nuclear compression by AFM.

A–D HeLa cells were transfected with AP1 GFP reporter or TEAD GFP reporter and plated on poly-L-lysine surface for 1 h. Then, atomic force microscopy was used to apply compressive force on the nucleus. After positioning the cantilever above the nucleus, constant height mode was used (target height of 3 µm below the surface) and maintained for 120 min. (c) Scale bar = 20 µm. Data are presented as mean ± s.e.m. (n = 12 minimum, ****P < 0.001 t-test).

Figure EV3. Nuclear shape regulates transcription factor activity.

A Membrane spot localization of the 15 TFs regulated by nuclear shape.
B Raw membranes of Affymetrix protein–DNA combo array kit after exposition to the ChemiDoc (Abcam) apparatus.
C mRNA expression for corresponding TF's target genes. Using the TRRUST database, we selected genes known to be positively regulated by these 15 TFs and we analyzed their mRNA expression: SPP1 gene for FOXD3; SLC4A9 gene for FOXI1; PCSK9 gene for HNF-4; AHRR gene for AHR; SOD2 gene for NFE2L2; BAMB1 gene for FOXF2; HBB gene for NFE2L2; PPAR gene for HIF1A; TGFB1 gene for USF1; and FAM89B gene for NFIA. Only genes with increase in mRNA expression superior to 1.5-fold change in response to nuclear flattening were selected. Data are presented as mean ± s.e.m. (n = 4 minimum, t-test *P < 0.5; ns: not significant).
D Protein–Protein Interaction Network reconstruction and analysis of the AP1, TEAD, SP1, and PPAR.
E Cell height was measured using AFM in cells cultured on soft or stiff matrix. Data are presented as mean ± s.e.m. (n = 15 minimum, ****P < 0.001 t-test).
F AP1 activity was measured in cells depleted or not for c-Jun in Ctrl, Blebb, and Blebb + AP conditions. Data are presented as mean ± s.e.m. (n = 5, ****P < 0.001 one-way ANOVA—Tukey's multiple comparisons post-test)
G Percentage of EdU-positive cells for cells depleted or not for c-Jun and YAP. Data are presented as mean ± s.e.m. (n = 4 independent experiments with at least 60,000 events for each condition). Data have been generated from two independent experiments using two different siRNAs targeting c-Jun and YAP. ***P < 0.01 one-way ANOVA—Tukey's multiple comparisons post-test.
H TEAD activity was analyzed in HeLa cells co-transfected with a Renilla plasmid as a luciferase reporter plasmid controlled by the TEAD-responsive promoter, and with a Renilla plasmid as a gene reporter; HeLa cells were depleted or not for SUN1 and SUN2. Data are presented as mean ± s.e.m. (n = 6, t-test *P < 0.5)
I AP1 activity was measured in cells depleted or not for SUN1 and SUN2. Data are presented as mean ± s.e.m. (n = 5, ****P < 0.001 t-test)
J mRNA expression for corresponding TF's target genes for HeLa cells depleted or not for SUN1 and SUN2. Data are presented as mean ± s.e.m. (n = 4, t-test *P < 0.5; **P < 0.1; ***P < 0.01).

© 2019 The Authors

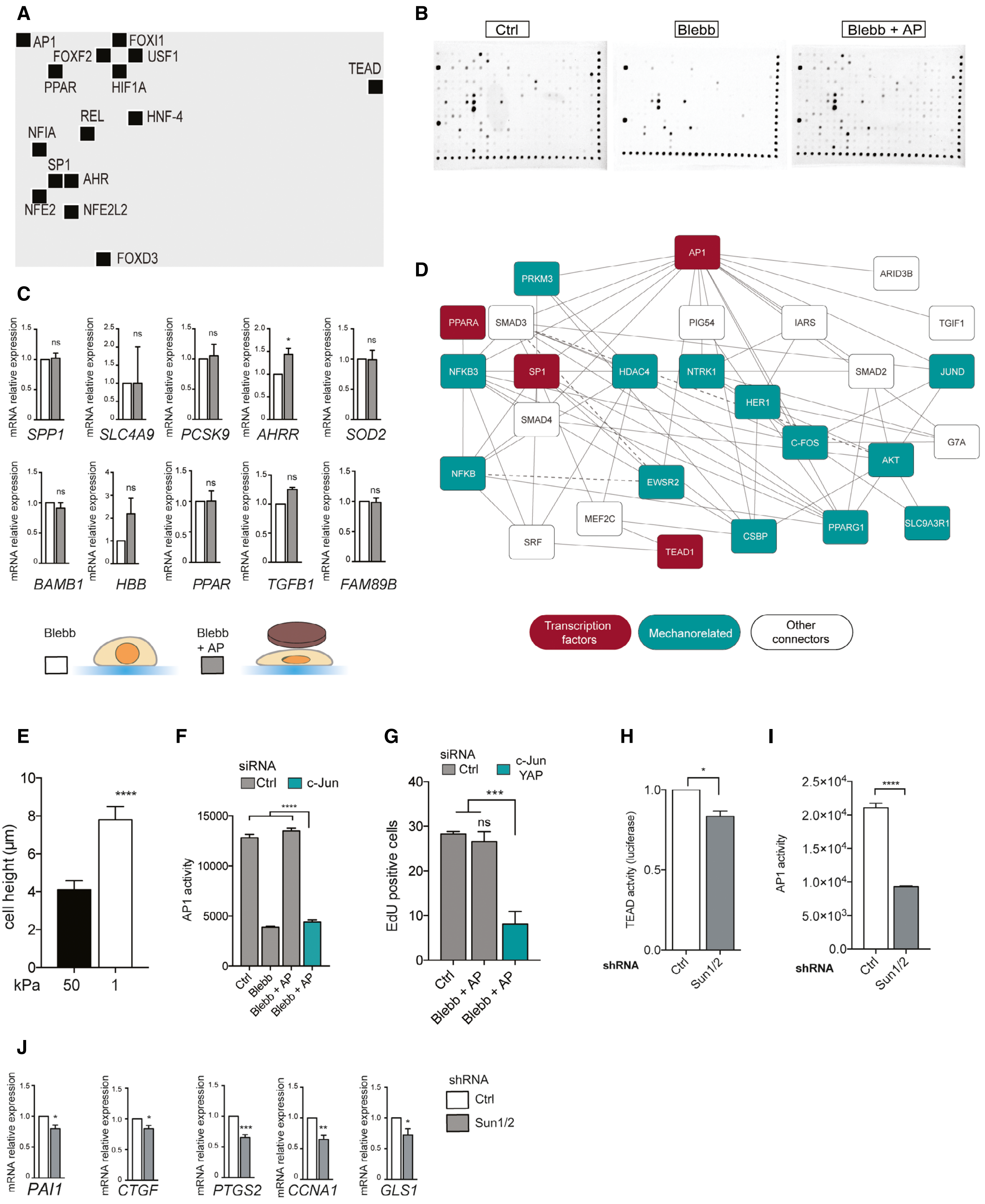

Figure EV3.

© 2019 The Authors

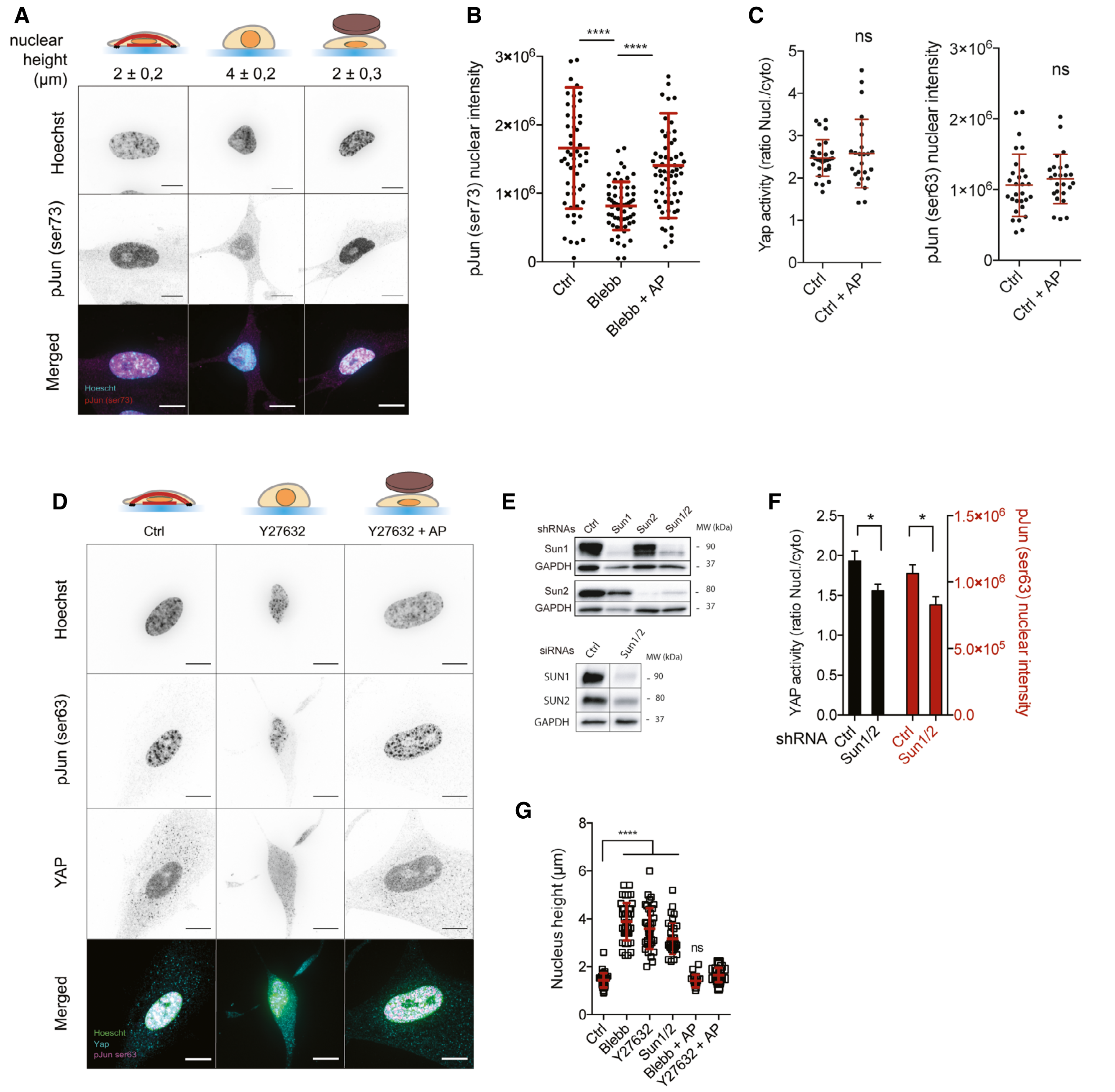

Figure EV4. Nuclear flattening is sufficient to activate c-Jun and YAP independently of actomyosin contractility.

A Representative cells stained for p-Jun Ser73 (magenta) and for nucleus (cyan) in Ctrl, Blebb, and Blebb + AP conditions. Scale bar = 10 µm. Nuclear heights were measured using Hoechst staining.

B Corresponding quantifications of p-Jun Ser73 nuclear intensity. Data are presented as mean ± s.e.m. (n = 58 minimum from two independent experiments, ****P < 0.001 one-way ANOVA—Tukey's multiple comparisons post-test).

C Quantifications of YAP activity (nucleo-cytoplasmic ratio) and p-Jun Ser63 nuclear intensity in Ctrl and Ctrl + AP conditions. Data are presented as mean ± s.e.m. (n = 25 minimum, ns: not significant, t-test).

D Representative cells stained for p-Jun Ser63 (magenta) and for DNA (cyan) in Ctrl, Y27632, and Y27632 + AP conditions. Scale bar = 10 µm.

E Immunoblots of SUN1, SUN2, and GADPH for MRC5 cells depleted or not for SUN1, SUN2, and SUN1 and SUN2 using siRNA or shRNA approaches.

F Quantifications of YAP activity (nucleo-cytoplasmic ratio) and p-Jun Ser63 nuclear intensity in cells depleted or not for SUN1 and SUN2. Data are presented as mean ± s.e.m. (n = 20 minimum, t-test *P < 0.5).

G Nucleus heights were measured using Hoechst staining in the indicated conditions. Data are presented as mean ± s.e.m. (n = 18 minimum, ****P < 0.001 one-way ANOVA—Tukey's multiple comparisons post-test).

© 2019 The Authors

Figure EV5. Molecular mechanisms mediating YAP and c-Jun activation in response to nuclear flattening.

A Representative cells stained for p-Jun Ser63 (magenta) and for DNA (cyan) in Ctrl, Blebb, and Blebb + AP conditions, treated or not with importazole. Scale bar = 10 μm.

B Quantifications of p- Jun Ser63 nuclear intensity in Ctrl, Blebb, and Blebb + AP conditions, treated or not with importazole. Data are presented as mean ± s.e.m. (n = 44 minimum from two independent experiments, ****$P < 0.001$ one-way ANOVA—Tukey's multiple comparisons post-test).

C Immunoblots of p-YAP (Ser127), YAP, and GFP for HeLa cells transfected with these following plasmids: Ctrl-GFP, LATS1 ^{WT}-GFP, LATS1T^{1079E}-GFP, LATS1^{T1079D}-GFP.

D Quantifications of YAP activity (nucleo-cytoplasmic ratio) in Ctrl, Blebb, and Blebb + AP conditions for cells transfected with GFP, LATS1-GFP, LATS1^{T1079E}-GFP, or LATS1^{T1079D}-GFP. Data are presented as mean ± s.e.m. (n = 18 minimum ****$P < 0.001$ one-way ANOVA—Tukey's multiple comparisons post-test).

E Nuclear heights were measured for cells transfected with GFP in Ctrl and Blebb conditions, and for those transfected with LATS1-GFP, LATS1^{T1079E}-GFP, LATS1^{T1079D}-GFP in Ctrl condition. Data are presented as mean ± s.e.m. (n = 18 minimum ****$P < 0.001$ one-way ANOVA—Tukey's multiple comparisons post-test).

F Immunoblots of lamin A/C and vinculin for MRC-5 cells depleted or not for lamin A/C.

G Quantification of YAP activity in Ctrl, Blebb, and Blebb + AP conditions for cells depleted or not for lamin A/C. Data are presented as mean ± s.e.m. (n = 48 minimum (TOP), n = 12 minimum (BOTTOM), ****$P < 0.001$, *$P < 0.05$ one-way ANOVA—Tukey's multiple comparisons post-test).

H Representative cells stained for p-Jun Ser63 (magenta), for YAP (cyan), and for DNA (green) treated with ATR/ATM inhibitor. Scale bar = 10 μm.

I Quantifications of p-Jun Ser63 nuclear intensity in Ctrl, Blebb, and Blebb + AP conditions for cells treated with an ATR/ATM inhibitor. Data are presented as mean ± s.e.m. (n = 30, ****$P < 0.001$ one-way ANOVA—Tukey's multiple comparisons post-test.).

▶

© 2019 The Authors

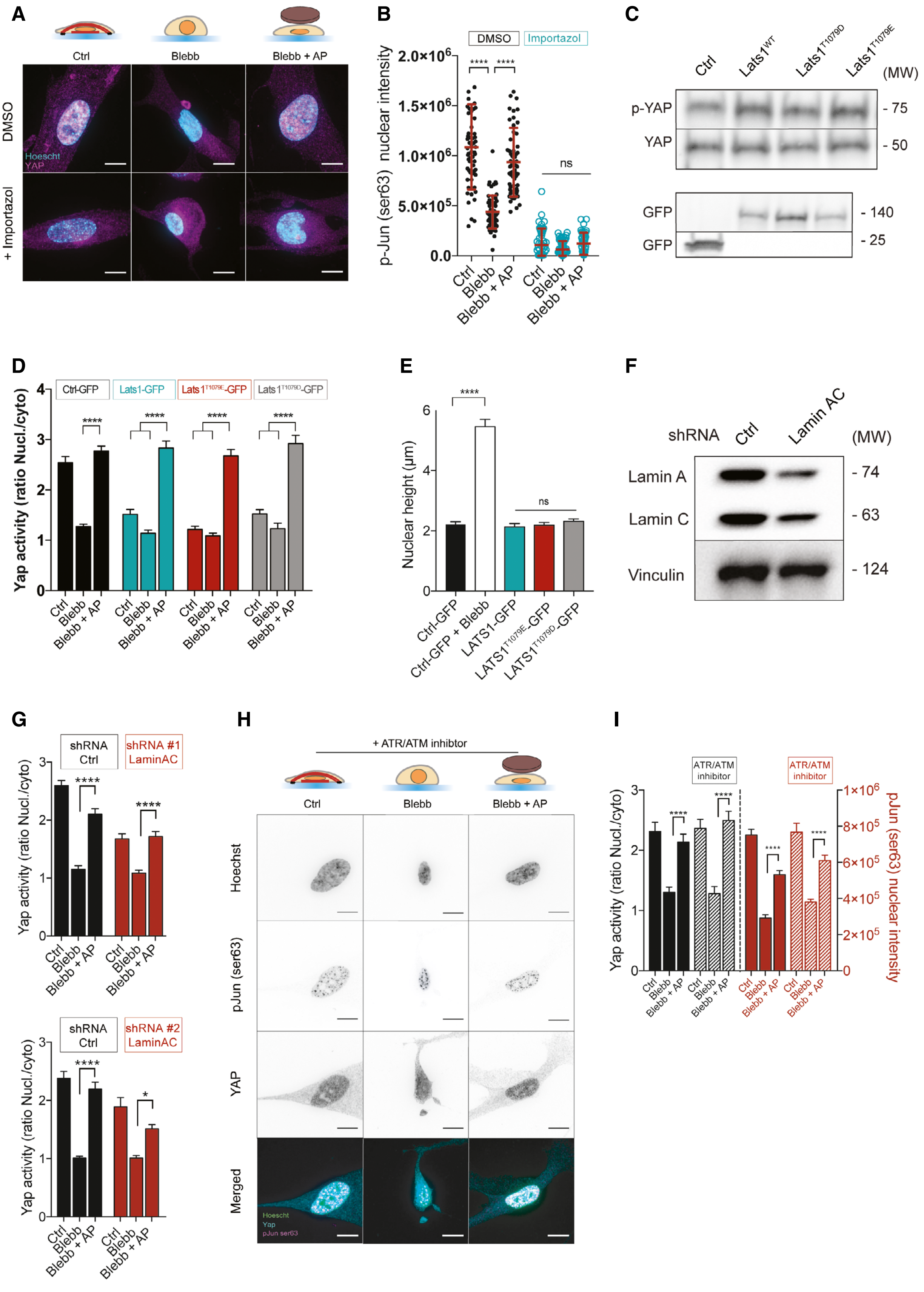
A
Ctrl
Blebb
Blebb + AP
DMSO
+ Importazol
Hoescht
YAP
B
DMSO
Importazol
p-Jun (ser63) nuclear intensity
ns
C
Ctrl
(MW)
p-YAP
YAP
GFP
- 75
- 50
- 140
- 25
D
Ctrl-GFP
Lats1-GFP
Yap activity (ratio Nucl./cyto)
E
Nuclear height (µm)
Ctrl-GFP
Ctrl-GFP + Blebb
LATS1-GFP
F
shRNA
Ctrl
Lamin AC
(MW)
Lamin A
Lamin C
Vinculin
- 74
- 63
- 124
G
shRNA Ctrl
shRNA #1 LaminAC
shRNA #2 LaminAC
H
+ ATR/ATM inhibtor
Hoechst
pJun (ser63)
YAP
Merged
I
ATR/ATM inhibitor
pJun (ser63) nuclear intensity

Figure EV5.
