# Supplementary material for: Nuclear envelope deformation controls cell cycle progression in response to mechanical force
Source: EMBO Rep. 2019 Aug 1;20(9):e48084. doi: 10.15252/embr.201948084 (PMC6726894; doi:10.15252/embr.201948084)
Supplement: Supplementary file 2 — Table EV1 [file EMBR-20-e48084-s002.pdf]

Table EV1. Antibody listing

Name	Supplier	Ref	Concentration	Method
Cdt1	Abcam	ab236152	1/750	IF
GADPH	Thermofisher	MA5-15738	1/2000	WB
Geminin	Abcam	104306	1/250	IF
GFP	Santa Cruz	sc9996	1/250	WB
JNK	Cell signaling	9252	1/750	WB
p-JNK (Thr183/Tyr185)	Cell signaling	9251	1/750	WB
p-c-Jun (ser73)	Abcam	ab30620	1/250	IF
p-c-Jun (ser63)	Cell signaling	2361	1/250	IF
KIBRA	Cell signaling	8774	1/1000	WB
Lamin A	Abcam	Ab26300	1/2000	IF
LATS1	Cell signaling	3477	1/1000	WB
p-LATS1 (Thr1079)	Cell signaling	8654	1/1000	WB
MST1	Cell signaling	3682	1/1000	WB
p-MST1 (Thr183)/MST2 (Thr180)	Cell signaling	3681	1/1000	WB
PTPN14	Cell signaling	13808	1/1000	WB
Sun1	Abcam	Ab124770	1/750	WB
Sun2	Abcam	Ab124916	1/750	WB
paxillin	Abcam	32084	1/250	IF
paxillin	Abcam	32084	1/750	WB
p-paxillin (Y118)	Cell signaling	2541	1/750	WB
Vinculin	Sigma	V9131	1/2000	WB
Yap	Santa Cruz	Sc101199	1/200	IF
Yap	Cell signaling	8418	1/1000	WB
p-Yap (ser127)	Cell signaling	13008	1/1000	WB
Alexa 568 goat anti Mouse	Invitrogen	A11004	1/500	IF
Alexa 488 goat anti Rabbit	Invitrogen	A11008	1/500	IF
Alexa Fluor 647 Phalloidin	Invitrogen	A22287	1/50	IF
