# Supplementary material for: Nuclear envelope deformation controls cell cycle progression in response to mechanical force
Source: EMBO Rep. 2019 Aug 1;20(9):e48084. doi: 10.15252/embr.201948084 (PMC6726894; doi:10.15252/embr.201948084)
Supplement: Supplementary file 3 — Table EV2 [file EMBR-20-e48084-s003.pdf]

Table EV2: Taqman gene primers ID

	genes	Assay ID
1	*gapdh*	Hs02786624_g1
2	*RPS6*	Hs04195024_g1
3	*18s*	Hs03003631_g1
4	*SERPINE1*	Hs00167155_m1
5	*CCND1*	Hs00765553_m1
6	*ITGB2*	Hs00164957_m1
7	*EGR1*	Hs00152928_m1
8	*CTGF*	Hs00170014_m1
9	*PTGS2*	Hs00153133_m1
10	*TGFB1*	Hs00998133_m1
11	*TGFB2*	Hs00234244_m1
12	*MMP2*	Hs01548727_m1
13	*MMP7*	Hs01042796_m1
14	*CDKN1A*	Hs00355782_m1
15	*MYC*	Hs00153408_m1
16	*IL6*	Hs00174131_m1
17	*CREM*	Hs01582003_g1
18	*CXCR4*	Hs00607978_s1
19	*Cyclin A1*	Hs00171105_m1
20	*gls1*	Hs01014020_m1
21	*vinculin*	Hs00419715_m1
22	*SRF*	Hs01065256_m1
23	*sdpr*	Hs00190538_m1
24	*FOS*	Hs04194186_s1
25	*gata6*	Hs00232018_m1
26	*FAM89B*	Hs00822402_g1
27	*Hbb*	Hs00758889_s1
28	*CETP*	Hs00163942_m1
29	*LPL*	Hs00173425_m1
30	*Coll11A2*	Hs00899176_m1
31	*AHRR*	Hs01005075_m1
32	*SOD2*	Hs00167309_m1
33	*BAMBI*	Hs03044164_m1
34	*CXCL12*	Hs03676656_mH
35	*SPP1*	Hs00959010_m1
36	*RARB*	Hs00977140_m1
37	*Slc4a9*	Hs00324675_m1
38	*Col1A1*	Hs00164004_m1
39	*Glut1*	Hs00892681_m1
40	*Fabp7*	Hs00361424_g1
41	*Sod1*	Hs00533490_m1
42	*PCSK9*	Hs00545399_m1
43	*CXCR4*	Hs00607978_s1
44	*sp1*	Hs00916521_m1
45	*c-Jun*	Hs01103582_s1
46	*hnf4*	Hs00230853_m1
47	*PPAR*	Hs00947536_m1
48	*AHR*	Hs00169233_m1
