# Supplementary figures and images for: Nuclear envelope deformation controls cell cycle progression in response to mechanical force
Source: EMBO Rep. 2019 Aug 1;20(9):e48084. doi: 10.15252/embr.201948084 (PMC6726894; doi:10.15252/embr.201948084)

First panel

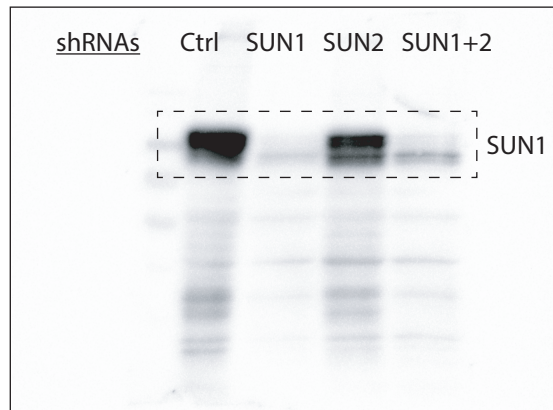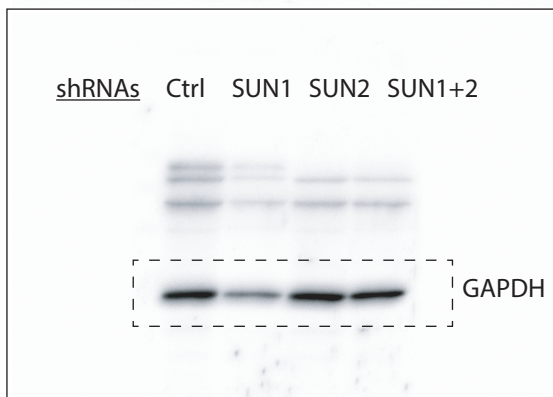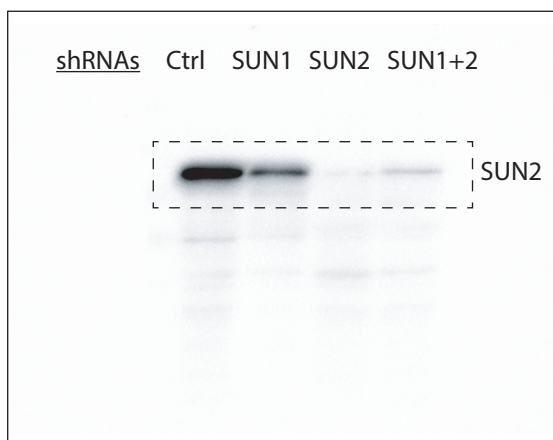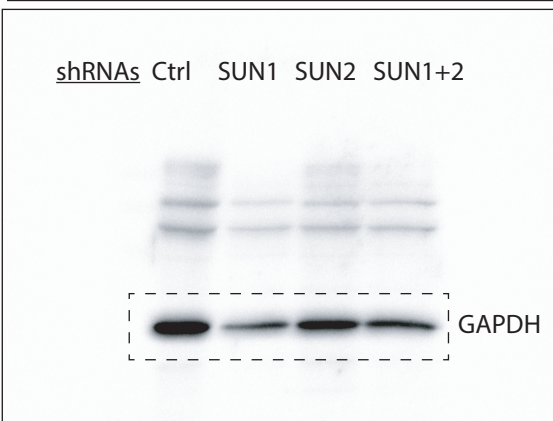

Second panel

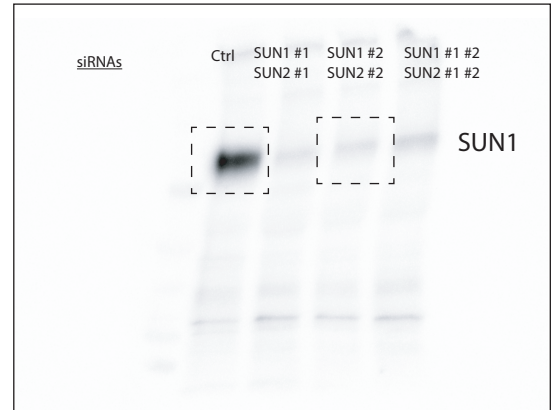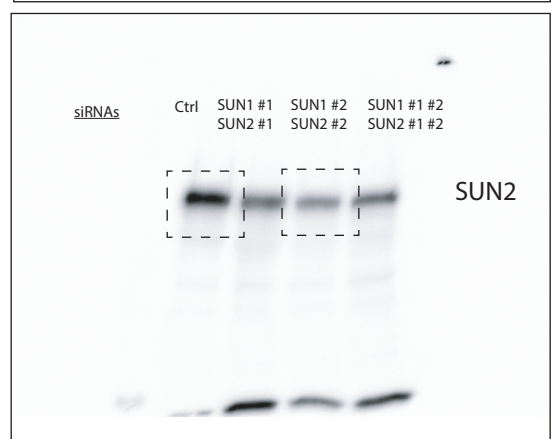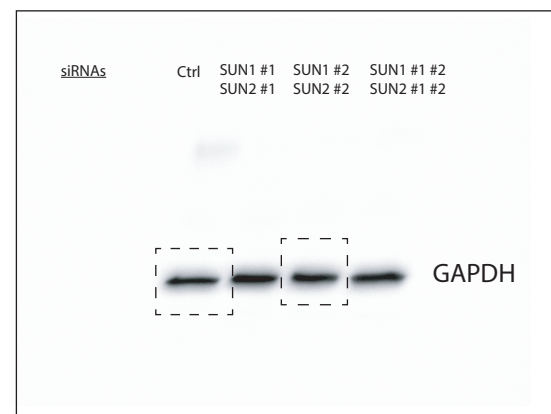

Supplement: Supplementary file 6 — Source Data for Appendix [file EMBR-20-e48084-s006.zip › embr201948084-sup-0006-SDataEV/embr201948084-sup-0006-SDataEV.pdf]
